# The Relationship Between the Gross Motor Function Classification System, Functional Mobility Scale, Observational Gait Scale, and the Amsterdam Gait Classification in Children with Cerebral Palsy During Long-Term Treatment with Botulinum Toxin Injections and Combined Integrated, Intensive Rehabilitation

**DOI:** 10.3390/toxins18020100

**Published:** 2026-02-15

**Authors:** Weronika Pyrzanowska, Magdalena Chrościńska-Krawczyk, Nigar Dursun, Marcin Bonikowski

**Affiliations:** 1 Health Sciences Department, The Maria Grzegorzewska University, 02-353 Warsaw, Poland; wpyrzanowska@aps.edu.pl; 2 Department of Children’s Neurology, University Children’s Hospital, 20-093 Lublin, Poland; 3 Department of Physical Medicine and Rehabilitation, Faculty of Medicine, Kocaeli University, 41001 Kocaeli, Turkey

**Keywords:** cerebral palsy, intensive, integrated rehabilitation, botulinum toxin, mobility, gait pattern

## Abstract

Patients with cerebral palsy (CP) experience complex gait disorders that change with age, leading to reduced activity and social participation. This study aimed to analyse how gait patterns developed over five years and to examine the relationships between the Observational Gait Scale (OGS), Amsterdam Gait Classification (AGC), Gross Motor Function Classification System (GMFCS), and the Functional Mobility Scale (FMS) at 5 and 50 m (FMS 5/50) during treatment. This retrospective, single-centre observational study involved annual assessments over a five-year period, which were analysed. Patients underwent a rehabilitation programme including physiotherapy, orthotics, multilevel botulinum toxin type A injections (BoNT-A), and serial casting. Data regarding BoNT-A treatment, casting, physiotherapy, orthoses, GMFCS levels, and FMS 5/50 scores were obtained from medical records. OGS and AGC were evaluated through two-plane clinical video recordings conducted in the same gait laboratory for all children. A cohort of 200 pediatric subjects (120 boys and 80 girls) diagnosed with bilateral cerebral palsy, predominantly classified as GMFCS II (48%) and III (36%), was analyzed. The average initial age was 32.23 months (±6.96), and GMFCS levels improved in 33. 5% of children and worsened in 2% (*p* < 0.001). Improvements were observed in 50% of children with GMFCS III and 40% with GMFCS IV levels. FMS 5 and 50 improved by 54% and 52%, respectively. OGS scores showed improvement in 74% and 76% of patients, respectively, while deterioration was observed in 5% and 7% for the right and left lower limbs, respectively. Most changes in OGS scores ranged from 1 to 4 points. A negative correlation was found between OGS and GMFCS (*p* < 0.001), and a positive correlation was found between OGS scores and FMS 5 and FMS 50 (*p* < 0.001). Additionally, significant relationships were identified between AGC and GMFCS, as well as FMS at 5 and 50 m. Complex gait disorders identified by the AGC are associated with higher GMFCS E&R scores and lower FMS scores. During the five-year follow-up, relationships were observed among GMFCS, FMS, OGS, and AGC. Our findings indicate that integrated treatment has a positive effect on functional mobility and gait patterns in patients with CP.

## 1. Introduction

Cerebral palsy (CP) is defined as a group of permanent movement and posture development disorders that result in activity limitations, caused by nonprogressive disturbances in the developing fetal or infant brain. [1]. A majority of patients with CP experience spasticity that disrupts motor function [2]. Spasticity frequently co-occurs with weakness, particularly in the lower-limb muscles, which can lead to abnormal gait patterns and potentially cause secondary musculoskeletal deformities. These can further increase the patient’s functional impairment [3]. Over the last thirty years, botulinum toxin type A (BoNT-A) has become a key treatment for hypertonia in children with CP. It promotes maximum developmental potential and helps prevent secondary complications [4]. The most significant advancement in managing children with CP was the adoption of multi-level BoNT-A injections within a comprehensive rehabilitation plan. This plan includes physiotherapy and orthotic interventions, which are considered essential components alongside other treatment options [3,5,6]. Such an integrated approach yields quantifiable outcomes and alters the natural progression of the disease [7,8]. The question regarding the long-term effects on gait remains relevant [4]. This study aimed to analyze how gait patterns developed over five years and to examine the relationships between the gait problems assessed by Observational Gait Scale (OGS) and Amsterdam Gait Classification (AGC) and mobility level described by Gross Motor Function Classification System Expanded and Revised (GMFCS E&R), and the Functional Mobility Scale (FMS) at 5 and 50 m (FMS 5/50) during treatment.

## 2. Results

The research cohort comprised 120 male and 80 female children, with ages ranging from 24 to 46 months. The mean age was 32.23 months (±6.96). Prior to the intervention, 6 children (3%) were classified at GMFCS level I, 96 (48%) at GMFCS level II, and 72 (36%) and 26 (13%) at GMFCS levels III and IV, respectively. All participants received Botulinum Toxin A (BoNT-A) injections and engaged in individualised physiotherapy regimens, as delineated in the Materials and Methods Section.

In the cases of 167 patients (representing 83.5%) and 166 patients (representing 83%) concerning the right and left lower limbs respectively, the assessment according to the Amsterdam Gait Classification during the stance phase showed no change (Table 1). An increase was observed in the number of patients demonstrating walking with an extended knee joint and complete foot contact, rising from 13 patients (6.5%) to 28 patients (14%) for the right lower limb, and from 14 patients (7%) to 27 patients (13.5%) for the left lower limb. The number of patients walking with an extended knee and a raised heel during the stance phase decreased from 132 (66%) to 106 (53%). A flexion pattern, classified as types 4 and 5, was identified in 55 patients (27.5%) and 54 patients (27%) during the initial assessment, in the left and right lower limbs, respectively. During subsequent assessments, this increased to 66 patients (33%) and 67 patients (33.5%), with an overall increase of 8 patients (4%) exhibiting gait type 5. No statistically significant difference was observed between the first and sixth assessments in gait pattern during the stance phase.

A noteworthy alteration in motor function was observed, with 67 children (33.5%) demonstrating an improvement in their GMFCS level (*p* < 0.001), whereas only 4 children (2%) exhibited regression. The frequency of improvement was especially significant among those initially classified as GMFCS level III (50%) and level IV (40%). FMS 5 and 50 m showed improvement in 54% and 52.5% of children, respectively. A significant increase in OGS values was observed from the initial to the sixth assessment (*p* < 0.001) across both legs. This progression in scores throughout the follow-up period was statistically significant (*p* < 0.001).

The right lower limb improved in 149 patients (74.5%), while the left lower limb improved in 153 patients (76.5%). Conversely, deterioration was observed in 17 cases (8.5%) for the right limb and in 14 cases (7%) for the left limb.

An analysis of the relationship between mobility level assessments, based on the GMFCS E&R classification, and the OGS score revealed correlations (*p* < 0.001) (Table 2). Throughout the observation period, the OGS score showed a decline consistent with the decrease in mobility levels indicated by the GMFCS E&R classification. Analysis of the relationship between gross motor function levels according to the GMFCS E&R classification and the OGS score showed correlations (*p* < 0.001) for patients classified at levels III and IV. Over the five-year observation period, the OGS score decreased as the assessment level based on the GMFCS E&R classification increased. Patients initially classified at level III had significantly lower OGS scores than those at level I, with a mean difference of 2.8 points, and an average annual decline of 0.314 points. For patients with GMFCS level IV, the difference was even greater (-3.68 points), but with a reduced and statistically insignificant annual decline of 0.128 points. Furthermore, analysis of the correlation between functional mobility assessments using the FMS, which measures distances of 5 and 50 m, and the OGS score revealed a relationship (*p* < 0.001), with higher scores noted on both scales. The differences in total OGS scores between patients with FMS 5 scores of 1 and 2, 4, 5, and 6 were approximately 1.8, 2.8, 4.1, and 4.9, respectively. Similarly, the differences in total OGS scores between patients with FMS 50 scores of 1 and 2, 4, 5, and 6 were approximately 1.7, 2.7, 4.0, and 5.0, respectively. Analysis of the relationship between the GMFCS E&R classification and the AGC assessment showed that patients with gait pattern type 2 during the stance phase had a significantly lower GMFCS E&R level than patients with gait pattern type 3 (*p* < 0.001), type 4 (*p* < 0.001), and type 5 (*p* < 0.01) during the stance phase. Analysis of the relationship between the assessment according to the FMS and the assessment according to the AGC revealed a statistically significant (*p* < 0.001) lower likelihood of achieving a higher score on the FMS over a 5-metre distance in patients with stance phase disorders. Achieving higher levels of functional mobility over a 5-metre distance was, on average, 5.2 times less likely (*p* < 0.001) for patients walking with an extended knee joint and no heel contact in MSt (AGC type 3) than for those walking with an extended knee joint without premature heel lift (AGC type 2). A similar pattern was observed for individuals with gait pattern type 4; in their case, the chance of improvement in FMS 5 was 1.9 times lower. For gait function over 50 m, significant associations were found for gait pattern type 3, with this chance decreasing fivefold (*p* < 0.001). No significant relationship was identified for patterns 4 and 5. Further analysis revealed that patients with the previously described swing-phase disorders [9], especially those with limitations in knee extension during terminal stance and foot drop, had a ten times lower likelihood (*p* < 0.001) of achieving a higher FMS score over 5 m. Regarding FMS 50, significant relationships were observed among patients exhibiting the aforementioned gait patterns during the swing phase, with an average 10.5-fold increase in risk (*p* < 0.001).

## 3. Discussion

This investigation extends previous findings [7], demonstrating improved ambulatory performance in most children. These gains were evident through positive changes in their GMFCS levels and FMS scores. The GMFCS level changed for 71 (35.5%) patients, of whom 67 (33.5%) improved by one grade and 4 (2%) deteriorated by one grade. Among patients with GMFCS level III who could walk on flat surfaces with a hand-held device before initiating treatment, the largest group showed improvement; 36 of these children (50%) achieved independent walking. The second-largest group exhibiting changes in GMFCS level comprised patients classified as level II. After a period of five years, approximately 25% of these patients demonstrated the ability to walk independently without limitations. In the smallest cohort of 26 patients classified as level IV before treatment, 9 (41%) showed changes. The study also showed a significant improvement in FMS (*p* < 0.001). Forty-four percent of patients demonstrated improvement at the 5 m distance, with 16% progressing by as many as four levels. The group of children with the highest score (6—indicating complete independence on all surfaces without the use of supporting devices or assistance) increased from 3 individuals (5%) in the initial assessment to 29 individuals (14%) in the final assessment. The number of patients rated at level 1, representing those who could only walk a few steps with assistance or using a walker, systematically decreased from 77 individuals (38%) initially to 7 individuals (5%) at the final assessment. Regarding the 50 m distance, 55.5% of children showed improvement, including 16% who improved by four levels. Similarly, there was a decrease in the number of patients graded at level 1, from 79 individuals (39%) to 7 individuals (5%). Forty children changed their levels after the first year of treatment, regardless of age, suggesting a potential role for the administered therapy. Over a span of five years, the number of patients reaching the highest FMS score (6) increased from 3 individuals (5%) to 26 individuals (13%). Notably, the improvement in mobility over 50 m is particularly important for functional purposes, as it allows patients to move freely within their environment, such as in educational settings. The main finding from the second publication [9] indicated that the treatment significantly improved gait kinematics. The results showed an increase in the overall OGS score over the years, with a statistically significant difference observed between the initial and the sixth measurements. After five years of follow-up, improvements (from 1 to 4 points) were seen in 74% and 76% of patients, respectively, while deterioration was noted in 8.5% and 7% of patients in the right and left lower limbs, respectively. Notably, the improvements remained stable and even increased over the five-year period.

One of objective of this study was to assess the impact of BoNT-A treatment, combined with individualized, goal-oriented therapy, on gait patterns, as measured by the AGC. The Amsterdam Gait Classification allows for the assessment of gait pattern disorders during the stance and swing phases. The basis of the classification is the description of movement in the upper ankle joint and the knee joint. For 167 (83.5%) and 166 (83%) patients, considering the right and left lower limbs respectively, the assessment according to AGC during the stance phase did not change. An increase was observed in the number of patients walking with a straightened knee joint and full foot contact with the ground, from 13 (6.5%) to 28 (14%) and from 14 (7%) to 27 (13.5%) for the right and left limbs. The number of individuals walking with a straightened knee joint and with the heel raised during mid-stance (MSt) decreased from 132 (66%) to 106 (53%). The flexion pattern (types 4 and 5) was observed in 55 (27.5%) and 54 (27%) individuals during the first examination, for the left and right lower limbs, and in 66 (33%) and 67 (33.5%) at the last examination, including an increase of 8 (4%) in the number of individuals with gait type 5. A statistically significant difference (*p* < 0.01) was demonstrated between the results of the first and sixth examinations concerning the gait pattern during the stance phase. The presented changes are similar to the results obtained from kinematic measurements [9] and indicate a beneficial effect of the applied therapy on the evolution of the gait pattern. The existing literature illustrates the progression of gait disorders with advancing age. O’Sullivan et al. [10] reviewed four studies and reported the progression of knee flexion deformity in 75 children with CP. The second objective of this study was to examine the relationships between the Observational Gait Scale, Amsterdam Gait Classification, Gross Motor Function Classification System, and the Functional Mobility Scale (at 5 and 50 m during treatment. Analysis of the relationship between the assessment of gross motor function level according to the GMFCS E&R classification and the OGS score demonstrated correlations for patients classified at levels III and IV (*p* < 0.001). Throughout the five-year observation period, the OGS score decreased as the assessment level according to the GMFCS E&R classification increased. Patients classified at level III achieved significantly lower OGS scores than those at level I, initially by an average of 2.8 points, with an average annual decrease of 0.314 points. For patients with GMFCS level IV, the difference was even larger (−3.68 points), but with a minimal and statistically insignificant annual decline (0.128 points). An analysis of the relationship between the assessment of functional mobility using the FMS for distances of 5 and 50 m and the OGS score indicated correlation (*p* < 0.001) of higher scores achieved by patients on both scales. A difference of approximately 5 points on the OGS (*p* < 0.001) was observed between patients with the lowest mobility level, reliant on a walker and external assistance, and those with the highest, who are autonomous across all surfaces, for both 5 and 50 m. The results presented confirm that both the level according to the GMFCS E&R classification and the score based on the FMS were influenced by the severity of existing gait pattern abnormalities, as assessed by OGS. An increase in the severity of these abnormalities correlates with decreased independence and poorer outcomes in the aforementioned classification and scales. The FMS facilitates the detection of changes in functional mobility over distances of 5 and 50 m, regardless of the stability of the GMFCS E&R level [11]. It is noteworthy that the FMS demonstrates greater sensitivity to changes; for example, patients classified at level III according to GMFCS E&R can improve their FMS scores by changing their strategies for functional mobility, which depends on the support of assistive devices. An example is using a cane instead of crutches, which results in an improved assessment from 3 to 4. The findings also confirmed that improvements in gait pattern, as measured by OGS, influence enhancements in GMFCS E&R. Similar observations are documented in studies discussing the outcomes of treatments following multi-level surgical procedures [11,12]. Analysis of the relationship between the assessment according to the GMFCS E&R classification and the AGC demonstrated a correlation between gait pattern during the stance phase and the level of mobility according to GMFCS E&R. The results of the conducted analyses indicate that the more complex the type of disorder, the greater the mobility limitations, and the higher the level of GMFCS E&R. This correlation is certainly multifactorial; however, a significant contributing cause is the substantial increase in load and energy expenditure associated with the severity of gait pattern disturbances, as well as a markedly weaker extensor apparatus in patients who move with flexion patterns, types 4 and 5 [13]. Similarly to our findings, Gonçalves et al. [14] discovered that individuals with bilateral spastic CP/GMFCS II and III exhibited alterations commonly associated with a crouch gait pattern (AGC IV and V).

Analysis of the relationship between FMS assessment and AGC assessment showed that patients with stance phase disorders had a significantly reduced (*p* < 0.001) chance of achieving a higher FMS score over a 5 m distance. Patients walking with an extended knee joint and no heel contact during the stance phase showed, on average, a 5.2 times greater reduction (*p* < 0.001) in functional mobility over a 5 m distance compared to those walking with an extended knee joint but without premature heel lift. A similar relationship regarding gait pattern type 2 was observed for individuals characterized by gait pattern type 4 during the stance phase; in their case, this chance was 1.9 times lower. In the case of gait function over 50 m, significant relationships were found for gait pattern type 3, with this chance decreasing fivefold (*p* < 0.001). No significant relationship was found for patterns 4 and 5. Hösl et al. [15] analyzed the relationship between kinematic gait abnormalities and everyday mobility in ambulatory children and youth with spastic CP. They found that the Gait Profile Score (GPS) correlates (*p* < 0.001) with indoor and outdoor mobility assessed with the MobQues47 Questionnaire, for both unilaterally and bilaterally involved children with CP. These findings align with our previously presented results.

The motor development observed in patients with cerebral palsy (natural history) is a result of natural development observed in all children and the consequences of the syndrome itself. There is always a possibility that long-term changes in patients are due not only to intervention but also to the natural course of development [16]. According to Rosenbaum et al. [17], the achievement of 90% of maximum gross motor development, as assessed using GMFM 66, occurs in patients with GMFCS E&R level III at the age of three years and eight months. Patients at levels II and I reach these milestones at approximately four years and five months, and four years and ten months, respectively. Because the age of the examined patients during the observation period significantly exceeded the range associated with the observed improvements, the cited publication cannot solely attribute these findings to the natural progression of gross motor development.

Literature reports indicate that the natural history of gait pattern and functional disorders tends to decline with age [18,19]. Patients who attain independent walking ability may subsequently lose this capability during adolescence [20]. The argument that lack of long-term changes after surgery in cerebral palsy indicates improvement is justified [12,21]. The degree of improvement documented in our cohort suggests a beneficial effect of the therapeutic interventions on gait functions and patterns in children diagnosed with CP. A significant factor that may have contributed to the notable improvement of the parameters discussed in the study was the supplementation of physiotherapy with botulinum toxin and appropriately selected orthopedic devices [22,23,24]. Furthermore, the implementation of an individually tailored therapy program, based on specific tasks aimed at achieving predefined objectives, appears to be particularly important [25]. Opheim et al. [26] demonstrated that adults with CP, especially those presenting bilateral spastic paresis and higher GMFCS E&R levels, have an increased risk of gait deterioration compared to individuals with milder impairments and better gross motor function. This increased risk is attributed to overexertion resulting from excessive strain on the musculoskeletal system, in an effort to meet social and environmental demands. Patients who are able to walk independently with the help of a handheld support device experience a significant improvement in quality of life, as children and adolescents classified at GMFCS level IV often need physical assistance or powered device support. These individuals typically stay seated for most of the day and can only walk short distances when using a walker under supervision. In light of the above, the rehabilitation process for children and adolescents with CP should focus on achieving the highest possible functional level that allows full participation in social and professional life.

A major advantage of this research is the uniformity of the participant pool, which consisted of young children with bilateral spastic cerebral palsy who underwent a unified intensive rehabilitation protocol at a single facility. The study’s robustness is further bolstered by the involvement of highly skilled therapists specifically trained in the assessments used and by the application of rigorous video-based re-evaluations. Conversely, the findings are constrained by a retrospective approach and the lack of a comparative control group, which could introduce bias. Furthermore, variability in home-based care serves as an additional limiting factor.

## 4. Conclusions

The results of the study align with previous research [7,9] and support the combined use of rehabilitation and BoNT-A injections in children with spastic bilateral cerebral palsy to enhance gross motor skills, mobility, and gait pattern and also to prevent gait deterioration caused by progressive structural changes. Relationships were identified between OGS, AGC, GMFCS, and FMS at 5 and 50 m. Complex gait disorders were associated with higher GMFCS E&R scores and lower FMS scores. Future research should consider incorporating validated, patient-centered outcome measures that focus on life satisfaction and quality of life. Additionally, it is crucial to assess whether improvements in mobility are sustained and persist over long periods, such as into adolescence or adulthood.

## 5. Material and Methods

Settings and Inclusion Criteria. Here are presented efficacy outcomes from a retrospective, single-centre observational study carried out at the Mazovian Neuropsychiatry Centre in Zagórze, Poland. The retrospective investigation comprised 200 consecutively treated children diagnosed with cerebral palsy (CP), who received botulinum toxin A injections in conjunction with an integrated rehabilitation program. The primary inclusion criteria encompassed: a diagnosis of spastic bilateral CP; an age range of 2 to 4 years at the onset of observation; GMFCS levels I to IV prior to initial injection; availability of gait assessment data and medical records spanning a follow-up period of five years; and gait assessment conducted prior to or at least three months subsequent to each BoNT-A injection. Exclusion criteria comprised other forms of CP, notably those with predominantly dyskinetic characteristics, GMFCS level V, and orthopedic procedures performed specifically due to CP.

BoNT-A Treatment and Rehabilitation Program.

During the follow-up period, all children received BoNT-A injections, engaged in individualised physiotherapy programs, and utilised ankle- foot orthoses (AFOS). BoNT-A was predominantly administered one to two times annually; most patients underwent between five and ten treatments during the observation period. The total doses per session ranged from 20 U/kg to 30 U/kg for Abobont- A/dysport (Ipsen Biopharm Ltd., Wrexham, UK) and from 10 U/kg to 20 U/kg for Onabont-A/Botox (Allergan and AbbVie, Irvine, CA, USA). In most cases, multilevel injections were administered to the hip flexors (iliopsoas, rectus femoris) and adductors (adductor longus, adductor brevis, gracilis), knee flexors (semimembranosus, semitendinosus), and foot plantar flexors (medial and lateral gastrocnemius), based on comprehensive assessments of muscle tone, range of motion, strength, and gait. Injections were administered under mild sedation with guidance from electrical stimulation or ultrasound. During the observation period, no patients received oral therapies for spasticity. An individualised physiotherapy protocol was developed for each child, with specific functional goals established to guide the therapeutic process. These goals, which emphasised functional mobility and daily activities, were formulated in collaboration with the children and their parents. The goals adhered to the five SMART criteria: specific, measurable, achievable, realistic, and time- bound [24,27]. The long- term objectives aimed to enhance the child’ s mobility, independence in daily activities, and social participation. Short- term objectives focused on improvements at the structural level, including passive and active range of motion, muscle strength, and selective motor control [28,29]. The frequency of intensive physiotherapy sessions varied from three to twelve weeks annually. The physiotherapy comprised both individual and group training sessions, each lasting approximately 120 min daily. Therapists employed analytical, functional, and task- oriented techniques in the treatment. All children continued rehabilitation within the community setting, attending sessions two to five times per week. All patients used rigid or semirigid AFOS complemented by appropriate footwear. Orthoses were individually adjusted based on ground reaction vector visualization. The primary aim of orthotic management was to improve gait parameters and movement patterns. Of the children, 169 (84.5%) wore AFOS for five to eight hours daily, while 31 (15.5%) utilised them for less than five hours per day.

### 5.1. Data Collection Procedure

Information regarding Botulinum Toxin Type A treatment, casting, physiotherapy, orthoses, GMFCS levels, and FMS ratings was collected from medical records. The Gross Motor Function Classification System (GMFCS) characterises the abilities and limitations of children and adolescents in gross motor function. According to established guidelines, children classified at level I are able to walk independently. Conversely, those assessed at level V rely on assistive devices and caregiver support for self-initiated movements, with particular emphasis on sitting, transfers, and mobility [30]. The Functional Mobility Scale (FMS) categorises functional mobility in children over distances of 5, 50, and 500 m, including the necessary assistive devices for each distance. These specific distances mainly serve an informational purpose, as environmental context is the most significant factor. Functional mobility across these distances reflects abilities within the home, school environment—including playgrounds—and outdoor settings such as shopping malls or streets. The mobility rating, on a scale of 6, indicates complete independence in navigating various surfaces and overcoming obstacles. When a child used sticks, crutches, or a walking frame, the FMS scores were 4, 3, and 2, respectively. A score of 1 characterised a child who used a wheelchair and required some caregiver assistance to climb steps [31]. The evaluation of OGS, AGC, and kinematics was carried out using two-plane clinical video recordings within the same gait laboratory for all paediatric subjects. The video recording procedures included: bright lighting with adequate contrast; cameras positioned perpendicular to the relevant planes of motion to minimise optical interference; a measurement pathway measuring 10 m in length and 2 m in width; the accessible measurement area, as displayed on the screen, measuring 2.5 m by 1.6 m; patient preparation involving exposure of the lower body and barefoot walking. The initial gait assessment, performed either before or at least three months after botulinum toxin type A injection in accordance with the individual injection schedule, was selected for evaluation at each follow-up year. Data analysed in this study originated from assessments conducted before or at least three months following BoNT-A injections, over a five-year period. A total of six gait assessments were chosen for each patient. All evaluators involved in the study were well-trained and possessed extensive expertise in gait analysis. The measurement results were presented using the Observation Gait Scale (OGS) [32]. Kinematic gait parameters were evaluated through two-plane video recordings with an integrated goniometer and corresponding software. The most representative gait cycle was analysed. The obtained measurement outcomes were presented using the Observation Gait Scale, the Amsterdam Gait Classification, and were supplemented with assessments of the knee and hip joint positions in TSt, the knee joint position in TSw, and foot clearance evaluation [7]. The results were subjected to statistical analysis. The Amsterdam Gait Classification helps in evaluating gait pattern disorders through video analysis. This system identifies five gait types observed during the stance phase in the sagittal plane [33]. It describes movement at the proximal ankle and knee joints. The AGC applies to both unilateral and bilateral gait deviations associated with cerebral palsy in pediatric patients. The Amsterdam Gait Classification categorizes five gait types based on knee angle and foot contact during mid-stance: Type 1: normal knee angle with full foot contact; Type 2: hyperextended knee with full foot contact; Type 3: hyperextended knee with incomplete foot contact, characterized as toe walking; Type 4: flexed knee with incomplete foot contact, also called toe walking; and Type 5: flexed knee with full foot contact, known as crouch gait.

### 5.2. Statistical Analysis

Due to multiple observations conducted on the same patient, the relationship between various scale scores concerning changes in gross motor skills, functional mobility, and gait disorders during follow-up was evaluated using mixed models. The fixed effects for these models are presented herein. Linear mixed models were employed to examine the association between OGS and FMS scores and the GMFCS score, with the OGS score serving as the response variable. Predictor variables included time, gender, and their interactions with these scores and the classification system, provided that the relationships between these variables and the endogenous variable were statistically significant. Additionally, the results of the GMFCS classification or the FMS score were incorporated as predictors. The potential association between GMFCS or FMS scores and the modified Amsterdam Gait Classification outcomes was analysed using mixed-effects ordered logistic regression and mixed-effects ordered probit regression models. The fixed-effects coefficients are reported for the mixed models discussed in this report. Coefficients in linear models indicate the estimated change in the response (endogenous) variable corresponding to a one-unit increase in the predictor (explanatory or exogenous) variable. For categorical explanatory variables, a value of zero was assigned if the category applies, and a value of one if not. The odds ratios derived from the ordinal logistic regression coefficients represent the multiplicative change in the likelihood of the outcome belonging to a superior category for every unit increase in the predictor variable. An odds ratio greater than one indicates increased odds; a value less than one indicates decreased odds or a negative impact of the predictor. In ordinal probit regression, positive coefficients reflect an increased probability that the response variable belongs to a superior category when a particular predictor category or a one-unit increase applies; negative coefficients indicate a decreased probability. The results of the mixed Poisson regression model were not included, as they were not statistically significant. The significance was deemed notable when the predictor in the model was a nominal variable, with one of the categories designated as a reference category against which the relationships between the other categories and the explanatory variable were compared. The significance level was set at α = 0.05; however, statistically significant results were also reported for levels α = 0.01 and α = 0.001. *p*-values indicating statistically significant results are emphasised in bold. All computations were conducted using the R statistical software package, version 3.6.0.

This study was retrospective and involved the analysis of anonymized medical records without active or passive participation from the individuals included. The classification and scale results were assessed collectively, maintaining anonymity. These procedures were part of routine practice, to which caregivers consented. No application was submitted to the Bioethics Committee for research and scientific use of the obtained results. The authors (MB, WP), who are staff members of the Neurorehabilitation Department, have previously treated the assessed group. The review of anonymized records and the reported outcomes did not influence routine clinical practice.

## Figures and Tables

**Table 1:** Comparison of gait pattern types according to the AGC during the first and sixth assessments.

Assessment Number														
	Gait Pattern Type	1R (N = 200)	1L (N = 200)	2R (N = 200)	2L (N = 200)	3R (N = 200)	3L (N = 200)	4R (N = 200)	4L (N = 200)	5R (N = 200)	5L (N = 200)	6R (N = 200)	6L (N = 200)	Test
Stance
	2	6.5% (N = 13)	7% (N = 14)	8% (N = 16)	8% (N = 16)	9.5% (N = 19)	8.5% (N = 17)	12% (N = 24)	12.5% (N = 25)	14% (N = 28)	14.5% (N = 29)	14% (N = 28)	13.5% (N = 27)	Chi square test (*p* > 0.05)
	3	66% (N = 132)	66% (N = 132)	64.5% (N = 129)	64.5% (N = 129)	65% (N = 130)	65% (N = 130)	58% (N = 116)	58% (N = 116)	52.5% (N = 105)	52.5% (N = 105)	53% (N = 106)	53% (N = 106)	
	4	25% (N = 50)	24.5% (N = 49)	23.5% (N = 47)	23.5% (N = 47)	22% (N = 44)	22.5% (N = 45)	25% (N = 50)	25% (N = 50)	27.5% (N = 55)	27% (N = 54)	26.5% (N = 53)	27% (N = 54)	
5	2.5% (N = 5)	2.5% (N = 5)	4% (N = 8)	4% (N = 8)	3.5% (N = 7)	4% (N = 8)	5% (N = 10)	4.5% (N = 9)	6% (N = 12)	6% (N = 12)	6.5% (N = 13)	6.5% (N = 13)		
Comparison of gait pattern types according to the AGC during the first and sixth assessments														
					Right limb					Left limb				
No change during the first and sixth assessments					83.5% (N = 167)					83% (N = 166)				
During the first assessment, the stance type was 4 or 5, and during the sixth assessment, it was 2 or 3					5.5% (N = 11)					5% (N = 10)				
During the first assessment, the stance type was 2 or 3, and during the sixth assessment, it was 4 or 5					11% (N = 22)					12% (N = 24)				

AGC—Amsterdam Gait Classification.

**Table 2:** Fixed effects for the linear mixed model explaining the relationship between the total OGS score and the GMFCS level.

	Coefficient	2.5%	97.5%	*p*-value
	11.2	10.6	11.8	<0.001
Year of assessment	0.203	0.0874	0.318	**<0.001**
GMFCS II ^1^	−0.369	−0.893	0.154	0.166
GMFCS III	−2.8	−3.37	−2.22	**<0.001**
GMFCS IV	−3.68	−4.42	−2.96	**<0.001**
Year of assessment: GMFCS II	0.0776	−0.0436	0.199	0.21
Year of assessment: GMFCS III	0.314	0.185	0.442	**<0.001**
Year of assessment: GMFCS IV	0.128	−0.0118	0.267	0.073
^1^ GMFCS level I is the reference category				
Fixed effects for a linear mixed model explaining the relationship between the total OGS score and the FMS score for a 5 m distance				
	coefficient	2.5%	97.5%	*p*-value
	7.653	7.231	8.076	<0.001
FMS 2 ^2^	1.833	1.563	2.102	**<0.001**
FMS 4	2.777	2.429	3.123	**<0.001**
FMS 5	4.079	3.769	4.395	**<0.001**
FMS 6	4.959	4.496	5.43	**<0.001**
^2^ FMS score 1is the reference category				
Fixed effects for a linear mixed model explaining the relationship between the total OGS score and the FMS score for a 50 m distance				
	coefficient	2.5%	97.5%	*p*-value
	7.725	7.303	8.149	<0.001
FMS 2 ^3^	1.705	1.438	1.972	**<0.001**
FMS 4	2.686	2.337	3.032	**<0.001**
FMS 5	4.027	3.715	4.344	**<0.001**
FMS 6	5.027	4.539	5.522	**<0.001**
^3^ FMS score 1is the reference category				
Fixed effects for the ordered probit regression model explaining the relationship between GMFCS level and AGC score				
	coefficient	2.5%	97.5%	*p*-value
Stance type 3 ^4^	0.728	0.33	1.127	**<0** **.** **001**
Stance type 4	0.778	0.232	1.324	**0** **.** **005**
Stance type 5	0.99	0.091	1.889	**0** **.** **031**
^4^ Type 2 according to the AGC is the reference category				
Fixed effects for a logistic regression model explaining the relationship between the FMS score for a 5 m distance and AGC score				
	odds ratio	2.5%	97.5%	*p*-value
Stance type 3	0.191	0.126	0.289	**<0.001**
Stance type 4	0.531	0.527	0.535	**<0.001**
Stance type 5	1.082	0.513	2.282	0.836
Fixed effects for a logistic regression model explaining the relationship between the FMS score for a 50 m distance and AGC score				
	coefficient	2.5%	97.5%	*p*-value
Stance type 3	0.197	0.108	0.36	**<0.001**
Stance type 4	0.594	0.292	1.208	0.15
Stance type 5	1.13	0.464	2.752	0.788

GMFCS—Gross Motor Function Classification System Expanded; FMS 5/50—Functional Mobility Scale at 5 and 50 m; OGS—Observational Gait Scale; AGC—Amsterdam Gait Classification.

## Data Availability

The original contributions presented in this study are included in the article. Further inquiries can be directed to the corresponding author.
